# Chikungunya: a decade of burden in the Americas

**DOI:** 10.1016/j.lana.2023.100673

**Published:** 2024-01-08

**Authors:** William M. de Souza, Guilherme S. Ribeiro, Shirlene T.S. de Lima, Ronaldo de Jesus, Filipe R.R. Moreira, Charles Whittaker, Maria Anice M. Sallum, Christine V.F. Carrington, Ester C. Sabino, Uriel Kitron, Nuno R. Faria, Scott C. Weaver

**Affiliations:** a Department of Microbiology, Immunology and Molecular Genetics, University of Kentucky, College of Medicine, Lexington, KY, USA; b Department of Microbiology and Immunology, University of Texas Medical Branch, Galveston, TX, USA; c World Reference Center for Emerging Viruses and Arboviruses, University of Texas Medical Branch, Galveston, TX, USA; d Global Virus Network, Baltimore, MD, USA; e Instituto Gonçalo Moniz, Fundação Oswaldo Cruz, Salvador, Bahia, Brazil; f Faculdade de Medicina da Bahia, Universidade Federal da Bahia, Salvador, Bahia, Brazil; g Laboratório Central de Saúde Pública do Ceará, Fortaleza, Ceará, Brazil; h Department of Genetics, Microbiology and Immunology, Institute of Biology, University of Campinas, Campinas, São Paulo, Brazil; i Coordenação Geral dos Laboratórios de Saúde Pública, Secretaria de Vigilância em Saúde, Ministério da Saúde, Brasília, Brazil; j Instituto de Ciências Biológicas, Universidade Federal de Minas Gerais, Belo Horizonte, Minas Gerais, Brazil; k Departamento de Genética, Universidade Federal do Rio de Janeiro, Rio de Janeiro, Rio de Janeiro, Brazil; l MRC Centre for Global Infectious Disease Analysis, Department of Infectious Disease Epidemiology, School of Public Health, Imperial College London, London, UK; m Departamento de Epidemiologia, Faculdade de Saúde Pública, Universidade de São Paulo, Brazil; n Department of Preclinical Sciences, Faculty of Medical Sciences, The University of the West Indies, St. Augustine, Republic of Trinidad and Tobago; o Instituto de Medicina Tropical, Faculdade de Medicina da Universidade de São Paulo, São Paulo, Brazil; p Departamento de Moléstias Infecciosas e Parasitárias, Faculdade de Medicina da Universidade de São Paulo, São Paulo, Brazil; q Department of Environmental Sciences, Emory University, Atlanta, GA, USA; r Department of Biology, University of Oxford, Oxford, UK; s Institute for Human Infections and Immunity, University of Texas Medical Branch, Galveston, TX, USA

**Keywords:** Chikungunya virus, Arbovirus, Mosquito-borne virus, Alphavirus, Americas

## Abstract

In the Americas, one decade following its emergence in 2013, chikungunya virus (CHIKV) continues to spread and cause epidemics across the region. To date, 3.7 million suspected and laboratory-confirmed chikungunya cases have been reported in 50 countries or territories in the Americas. Here, we outline the current status and epidemiological aspects of chikungunya in the Americas and discuss prospects for future research and public health strategies to combat CHIKV in the region.

## Introduction

Since the first autochthonous cases of chikungunya were reported in Saint Martin Island in December 2013, chikungunya virus (CHIKV) quickly spread and continued to cause epidemics throughout the Americas.^1^ CHIKV is a mosquito-borne alphavirus transmitted in human-amplified cycles by *Aedes* (*Stegomyia*) *aegypti* and *Ae.* (*Stegomyia*) *albopictus* mosquitoes, which are also vectors of dengue (DENV) and Zika (ZIKV), and yellow fever viruses.^2^ CHIKV can be classified into three major lineages: West African, East-Central-South-African (ECSA), and Asian.

Chikungunya is characterized by acute and chronic manifestations, typically including fever and polyarthralgia, which are often highly debilitating.^2^ Chikungunya can also result in severe manifestations, including neurological complications and death.^3^^,^^4^ Currently, chikungunya is a major public health problem in the Americas, where it causes a large economic burden due to direct and indirect costs. In November 2023, the first vaccine against chikungunya was approved by the U.S. Food and Drug Administration,^5^ but large immunization programs remain to be implemented, and specific antivirals remain unavailable to prevent and treat chikungunya.

In this viewpoint, we review and analyze the impact of CHIKV in the Americas since its introduction. First, we contextualize the epidemiology and patterns of CHIKV spread from 2013 to 2023 and provide hypotheses for the different epidemiological trajectories of CHIKV and ZIKV. Second, we review the evolutionary aspects of the CHIKV Asian-American and ECSA-American sub-lineages introduced into the Americas and evaluate genomic surveillance in the region. Third, we discuss the burden and severe manifestations caused by CHIKV infection, including the likely underestimated number of cases and fatal outcomes. Lastly, we discuss public health approaches with the potential to prevent, control, and eliminate chikungunya in the Americas.

## Epidemiology and patterns of chikungunya in the Americas

**Fig. 1:**
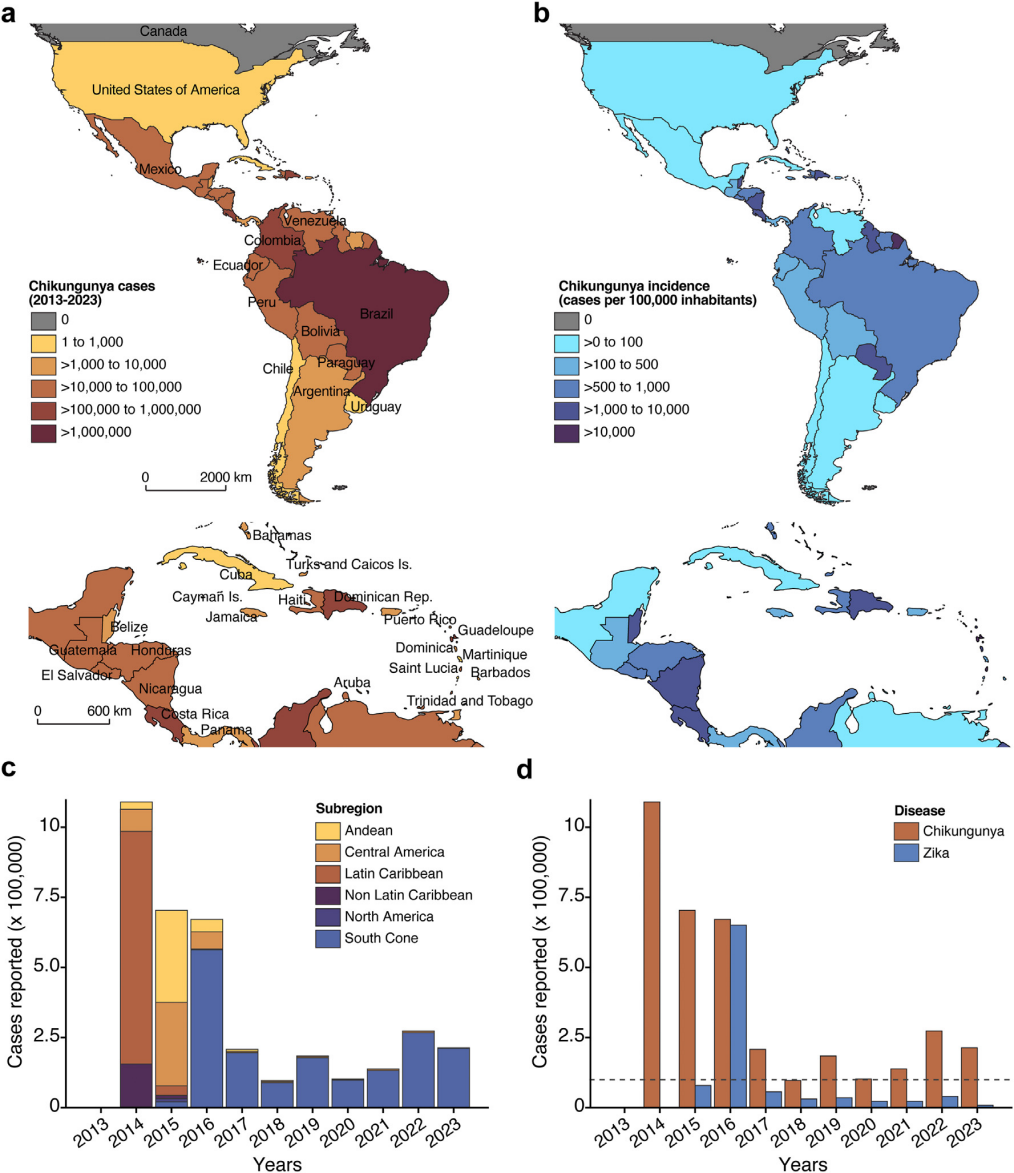
**Epidemiology of chikungunya in the Americas. (a)** The map shows cumulative chikungunya cases (i.e., suspected and laboratory-confirmed). In the Americas reported to the Pan American Health Organization (PAHO) from December 2013 to June 2023. A larger-scale map of Central America and the Caribbean region is shown at the bottom. **(b)** The map shows cumulative incidence of chikungunya cases (i.e., suspected and laboratory-confirmed) per 100,000 inhabitants per country in the Americas from 2013 to 2023. Incidence was calculated based on population data for 2023, estimated by an International Database from the United States Census Bureau. A larger-scale map of Central America and the Caribbean region is shown at the bottom. **(c)** Annual number of chikungunya cases (i.e., suspected and laboratory-confirmed). In the Americas reported to the PAHO from 2013 to 2023 by subregion (https://www3.paho.org/data/index.php/en/mnu-topics/chikv-en/550-chikv-weekly-en.html). In 2013, 109 chikungunya cases were reported in the Americas. **(d)** Annual number of chikungunya and Zika cases (i.e., suspected and laboratory-confirmed). in the Americas reported to PAHO from 2013 to 2023. The dashed line represents 100,000 cases reported in the Americas per year. The American subregions are presented based on the PAHO definition. Andean region: Bolivia, Colombia, Ecuador, Peru, and Venezuela. Central America region: Belize, Costa Rica, El Salvador, Guatemala, Honduras, Nicaragua, and Panama. Latin Caribbean region: Cuba, Dominican Republic, French Guiana, Guadeloupe, Guyana, Haiti, Martinique, Puerto Rico, and Saint Martin. Non-Latin Caribbean region: Anguilla, Antigua and Barbuda, Aruba, Bahamas, Barbados, Bonaire, Saint Eustatius and Saba, Cayman Islands, Curaçao, Dominica, Grenada, Jamaica, Montserrat, Saint Barthelemy, Saint Kitts and Nevis, Saint Lucia, Saint Vincent and the Grenadines, Sint Maarten, Suriname, Trinidad and Tobago, Turks and Caicos Islands, British Virgin Islands, and Virgin Islands (USA). North America region: Bermuda, Canada, Mexico, and the United States of America. South Cone region: Argentina, Brazil, Chile, Paraguay, and Uruguay.

Between December 2013 and June 2023, 3,684,554 chikungunya cases (suspected and laboratory-confirmed) were reported in 50 countries or territories in the Americas (Fig. 1a and b). In 2014, the largest chikungunya epidemics in the Americas were observed predominantly in the Latin Caribbean, followed by the Non-Latin Caribbean region. Subsequently, the Central American and Andean regions were the most affected in 2015 (Fig. 1c). Between 2014 and 2015, most territories or countries with chikungunya outbreaks in the Latin Caribbean reported one or two annual epidemic waves followed by a period with lower incidence or no cases. For example, Montserrat Island reported an explosive epidemic with a cumulative incidence of 85,493 cases per 100,000 inhabitants.^6^ Similarly, a serosurvey in Jamaica after the chikungunya epidemic in 2014–2015 showed a seroprevalence of 83.6%.^4^ Considering the lifelong immunity and protection elicited by CHIKV infection,^7^ this suggests a limited potential for CHIKV recirculation in these locations in the near future.

To date, chikungunya cases in North America represent 0.3% (12,172 of 3,684,554) of all reported cases in the Americas. Mexico reported 92.7% (12,034) of North American cases, with most cases in 2015 (Fig. 1a).^6^ However, considering the large immunologically CHIKV-naïve population in North America, combined with the projected expansion of *Ae. aegypti* and *Ae. albopictus* mosquito ranges due to climate change,^8^ autochthonous CHIKV transmission in North America could substantially increase in the future, particularly in the southern U.S., especially in states, such as Florida and Texas where *Ae. aegypti* is present and local CHIKV transmission has already been reported.^9^ Additionally, a small-scale serosurvey in Puente de Ixtla, Mexico, showed that 29.5% (114 of 387) of participants had CHIKV-specific IgG antibodies, suggesting greater than recognized CHIKV transmission in the region, given the small number of confirmed chikungunya cases reported in Mexico by the National Surveillance System.^10^ Situations like Puente de Ixtla are likely more common than reported and serosurvey studies are needed to better understand the dynamics of CHIKV transmission in the Americas and combat future epidemics.

## Brazil, the new epicenter of the chikungunya epidemic in the Americas

Brazil is the largest and most populous country in Latin America, with a large CHIKV-susceptible population, as well as a suitable climate, and abundant *Ae. aegypti* vector populations. CHIKV has circulated locally in Brazil since 2014, with early cases mostly restricted to the Northeast.^11^^,^^12^ Since 2016, Brazil is the epicenter of chikungunya epidemics in the Americas with 1,659,167 cases, the highest number reported in the region (Fig. 1a). In contrast to other countries and territories in the Americas, Brazil has been experiencing annual chikungunya epidemics.

In Brazil, spatial and temporal variation in chikungunya incidence appears to be driven by rapid, localized epidemics characterized by high rates of infection, followed by periods of lower incidence, probably driven at least in part by herd immunity from previous CHIKV infection.^11^ However, transmission at the country level is spatially heterogeneous, possibly due to the patchy landscape of CHIKV immunity, location-specific climates, differences in mosquito vectors, and exposure of people in different sociodemographic conditions. Although CHIKV serosurveys across Brazil remain scarce, 40.5% of municipalities have reported no chikungunya cases since 2014, suggesting that a significant proportion of the country's population remains susceptible or was infected but not captured by the national surveillance system.^11^ By sustaining prolonged CHIKV circulation, Brazil could become a focal viral source for spread to new geographic regions with large susceptible populations. Some regions may become more susceptible to outbreaks due to climate change, including North America, Europe, and other countries in the South Cone region of South America. Additionally, there is a risk of re-introduction to previously affected areas where pockets of susceptible populations may still exist. For example, Paraguay, a country with a population of 6.7 million, reported 85,889 chikungunya cases (annual cumulative incidence: 1154 per 100,000 inhabitants) in 2023.^6^ Likewise, Argentina reported 1336 chikungunya cases in 2023 after a hiatus of six years since its first epidemic in 2016.

## Different epidemiological trajectories of chikungunya and Zika in the Americas

Comparing the emergence and spread of CHIKV and ZIKV can provide critical insights into their epidemiology, as both viruses were introduced around the same time into immunologically naïve populations in the Americas, and both are transmitted by the same mosquito vector species vis human amplification.^12^^,^^13^ In September 2023, the number of reported chikungunya cases (n = 3,684,554) is 3.9 times higher than Zika cases (n = 949,567). CHIKV caused three major epidemics in the Americas, with 671,628–1,089,982 cases reported per year between 2014 and 2016, and over 97,000 cases per year from 2017 to 2023 (Fig. 1c and d). In contrast, ZIKV caused a single major epidemic with 650,867 cases reported in 2016, followed by a substantial decline in subsequent years. While the decline in Zika reports is probably due to herd immunity and its effect in limiting transmisison,^14^ small numbers of case reports may also be due to its higher rate of mild and inapparent infections, coupled with declining surveillance and milder signs and symptoms following typical infections, resulting in underreporting (Fig. 1d).

The divergent epidemiological trajectories of chikungunya and Zika epidemics may be partially explained by the very different clinical manifestations of their disease syndromes. Specifically, while CHIKV infections are usually symptomatic with a painful and often debilitating illness that restricts mobility, most ZIKV infections are mild or asymptomatic. Also, rates of symptomatic infections are estimated in the Americas at 51% for CHIKV and 20% for ZIKV.^15^^,^^16^ Since *Ae. aegypti* has a limited flight range,^17^ human mobility plays an important role in the long-distance spread of these viruses across susceptible populations. Thus, higher levels of disease-driven reductions in human mobility for CHIKV may reduce secondary infections arising from symptomatic cases, especially outside of the household, and limit the epidemics to local transmission pockets. Conversely, the higher proportion of asymptomatic ZIKV infections may have contributed to its faster and wider spread over longer geographic distances throughout the Americas and explain the fewer pockets of remaining naïve populations. Moreover, sexual transmission may be another epidemiological relevant route for ZIKV,^15^ but it has not been reported for CHIKV.

## Genomic surveillance of chikungunya virus in the Americas

Chikungunya and other mosquito-borne virus surveillance in the Americas is challenged by several factors, including reliance on passive surveillance, heterogeneous laboratory infrastructure, limited availability of skilled laboratory professionals and entomologists, and inequitable access to diagnosis. Moreover, the co-circulation of several different arboviral diseases with similar clinical manifestations (e.g., CHIKV, ZIKV, DENV, Mayaro, Venezuelan equine encephalitis, and Oropouche viruses) makes it challenging for clinicians to make an accurate diagnosis without laboratory testing. Underreporting and misdiagnosis hamper our collective understanding of the burden and dynamics of co-circulating arboviral diseases in the Americas as demonstrated previously for presumptive dengue cases.^18^^,^^19^ Therefore, there is an urgent need for countries in the Americas to implement and scale-up point-of-care diagnostics and cost-effective multiplex reverse transcription polymerase chain reaction tests to improve patient management and mosquito-borne disease control.^13^ This should be aligned with collaboration between local, national, and international health agencies and research institutions, with a wider goal to support capacity-strengthening initiatives, reduce inequalities in diagnostic and laboratory infrastructure, and strengthen and expand universal healthcare systems, which should be underpinned by equity, solidarity, and collective action to overcome social inequalities in the Americas.^20^^,^^21^

Traditional surveillance can be combined with genomic sequencing of mosquito-borne viruses to track the evolution and spread of newly introduced viruses or (re)-emerging virus lineages, and help guide the development of diagnostics, treatments, vaccines, and vector control strategies. As of August 2023, 1614 nearly complete genomes of CHIKV (i.e., ≥7000 nucleotides) from the Americas have been shared publicly on the National Center for Biotechnology Information (NCBI) GenBank database.^22^ This corresponds to around 44 genome sequences per 100,000 chikungunya cases reported in the region. Exceptionally, 94.3% (100 of 106) of cases reported in the U.S. were sequenced, mostly from autochthonous cases reported in Florida.

**Fig. 2:**
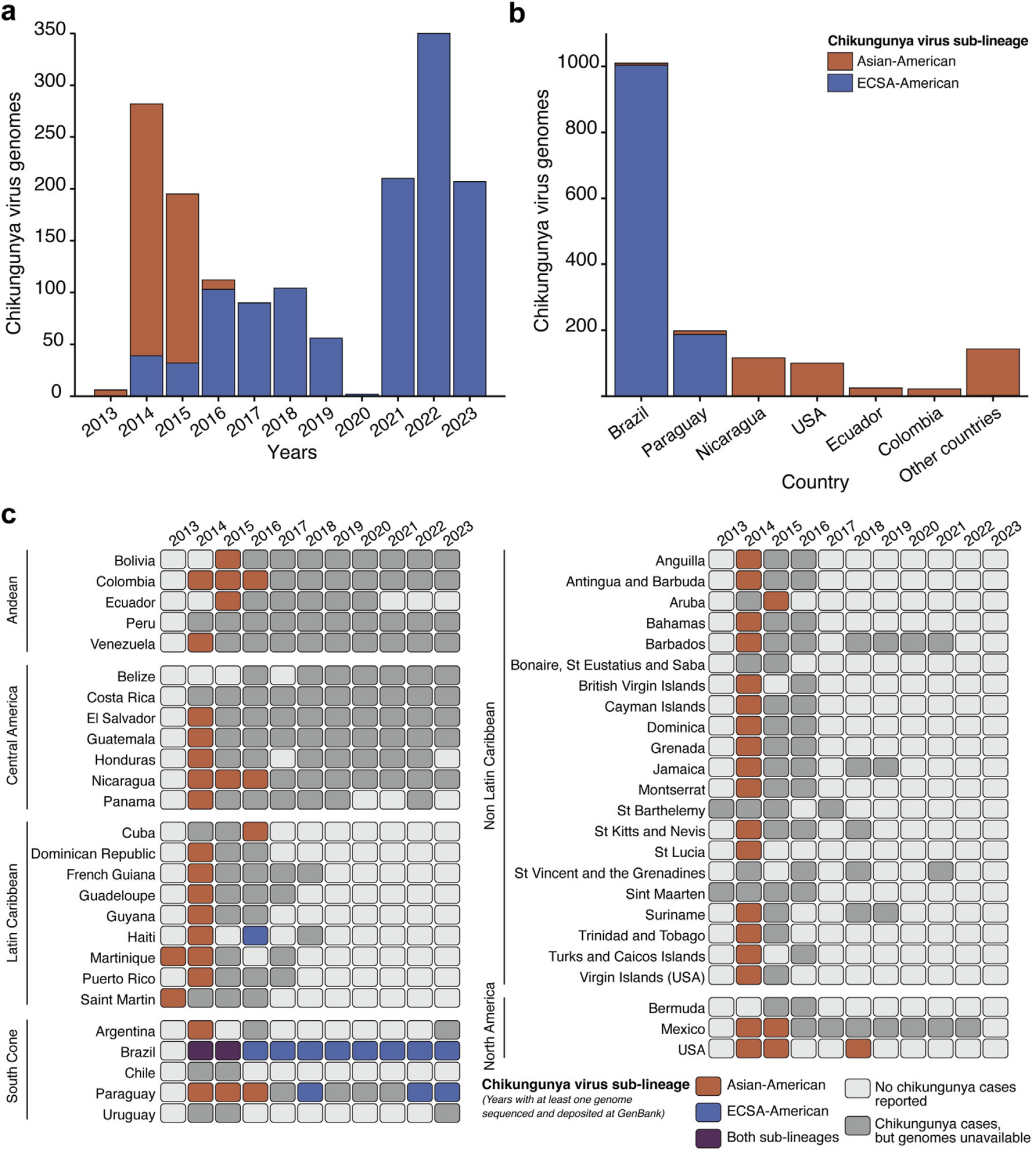
**Chikungunya virus lineage and genomic information**. **(a)** The number of chikungunya virus genomes sequenced and deposited into NCBI GenBank per year from 2013 to 2023, based on viral lineage. **(b)** The number of chikungunya virus genomes sequenced and deposited into NCBI GenBank per country based on chikungunya lineage up to 17 August 2023. **(c)** Spatiotemporal distribution of chikungunya virus lineages in countries and territories in the American subregions. The chikungunya lineage circulating was determined based on years with at least one genome sequenced and deposited at NCBI GenBank up to 17 August 2023. ECSA, East-Central-South-African lineage. USA, United States of America.

Most available CHIKV genomes are from samples collected between 2014 and 2015, the peak of the epidemic in the Americas, and between 2021 and 2023, during the recent epidemics in Brazil and Paraguay (Fig. 2a). To date, 62.6% of all CHIKV genomes shared on the NCBI GenBank are from Brazil, and 99.4% of these belong to the ECSA-American sub-lineage (Fig. 2b). Genomic data is currently unavailable for several countries with a high number of reported cases, such as Peru, Belize, and Costa Rica (Fig. 2c). Furthermore, despite the major 2014 and 2015 epidemics that affected the Caribbean, Central America, and Andean regions, there are limited genomic data available from these areas, where a small number of cases continues to be reported annually, particularly in Central America and the Andes regions.^6^

**Fig. 3:**
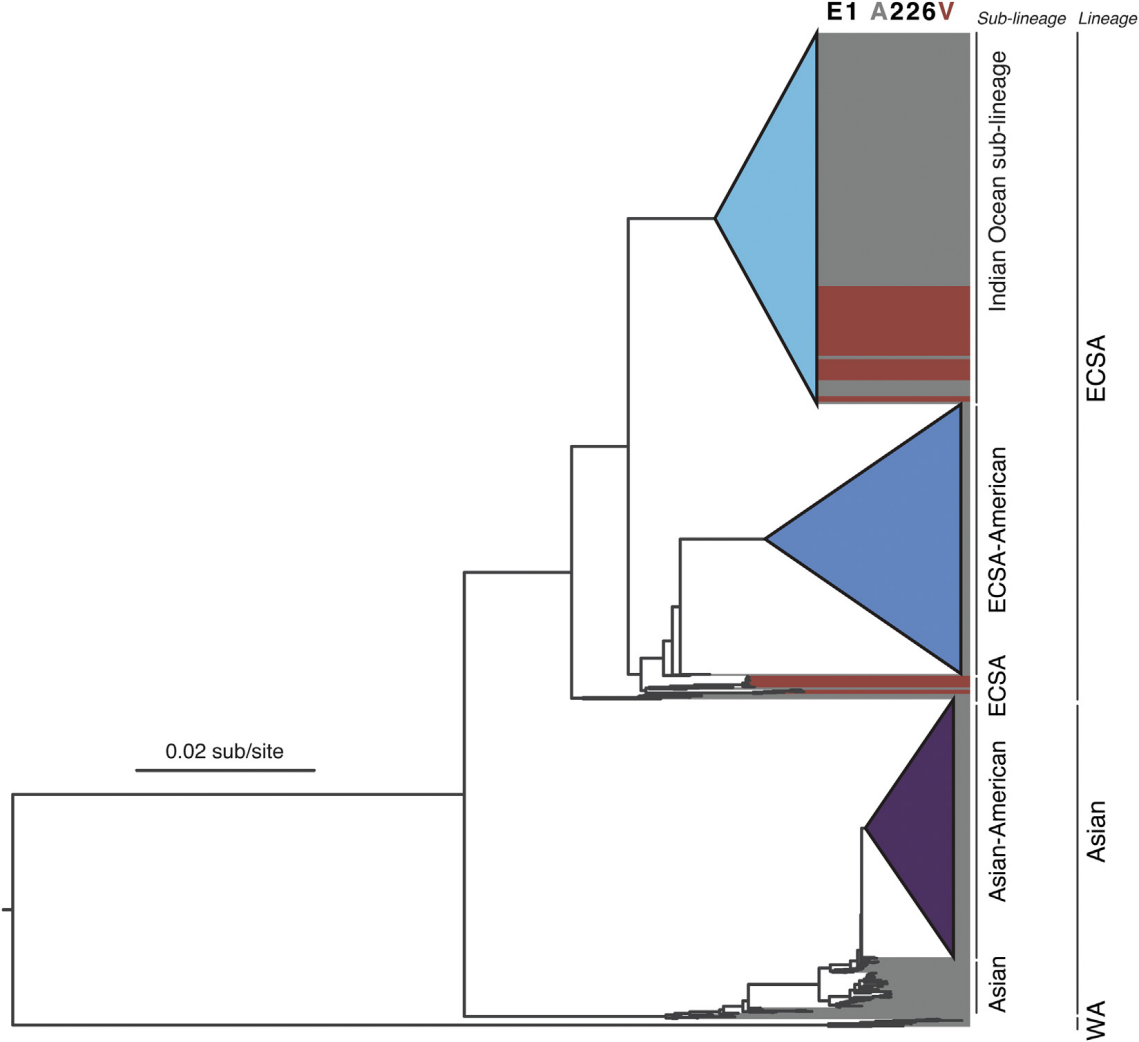
**Phylogenetic tree based on maximum likelihood method for the complete dataset of publicly available chikungunya virus genomes**. Branch lengths reflect genetic distances in substitutions per site. The chikungunya virus lineages and sub-lineages are indicated on the right side of the figure. The chikungunya virus sequences harboring the substitution of alanine (A) to valine (V) mutation at position 226 in the E1 envelope glycoprotein (E1-A226V) are indicated in purple. IOL, Indian Ocean sub-lineage. ECSA, East-Central-South-African. WA, West African. The Asian and ECSA lineage strains from the Americas are shown as Asian-American and ECSA-American sub-lineages, respectively.

Genomic surveillance can also contribute to the identification of lineage-specific mutations linked with increased transmission and vector adaptation. For example, the CHIKV epidemic in Reunion Island in 2005–2006 and subsequent outbreaks in the Indian Ocean islands, India, and China, were partly attributed to adaptive mutations in the envelope glycoproteins associated with increased infection and transmissibility by *Ae. albopictus* (Fig. 3).^23^^,^^24^ To date, these mutations, apparently subject to epistatic constraints,^24^ have not been described in CHIKV ECSA- or Asian-American sub-lineages, and experimental studies suggest that the most impactful of these mutations are not being selected due to epistatic constraints.^25^ However, improvement in genomic surveillance combined with experimental virology is needed to identify other adaptative mutations that could lead to better CHIKV fitness for mosquito transmission and prolonged or higher viremia in humans. Indeed, the infrastructure developed for virus genomic sequencing during the COVID-19 pandemic could be used to support CHIKV surveillance, as well as other endemic arboviruses in the Americas, such as DENV, ZIKV, and yellow fever virus.

## Genetic history of chikungunya virus in the Americas

RNA viruses evolve rapidly, and host–virus interactions leading to virus spread and diversification leave a measurable imprint on their genomes.^26^ The overall evolutionary rates of CHIKV have been estimated at around 4.3–8.4 × 10^−4^ nucleotide substitutions per site per year, translating to around 5–10 mutations being fixed at the population level per year across a genome of approximately 12,000 nucleotides.^27^ The Asian CHIKV lineage, which has circulated in Asia since at least the 1950s, caused the first autochthonous cases in the Americas and spread throughout the Caribbean islands and continental countries in 2013 and 2014 (Fig. 2a–c). To date, all sampled Asian lineage strains from the Americas (i.e., Asian-American sub-lineage) are monophyletic, supporting a point source introduction, probably from an infected traveler from Oceania or Southeast Asia (Fig. 3).^28^ The most recent common ancestor of the Asian-American sub-lineage was estimated around March 2013, suggesting that CHIKV was circulating in the Latin Caribbean region up to nine months before its first detection in December 2013 on Saint Martin Island.^29^

**Fig. 4:**
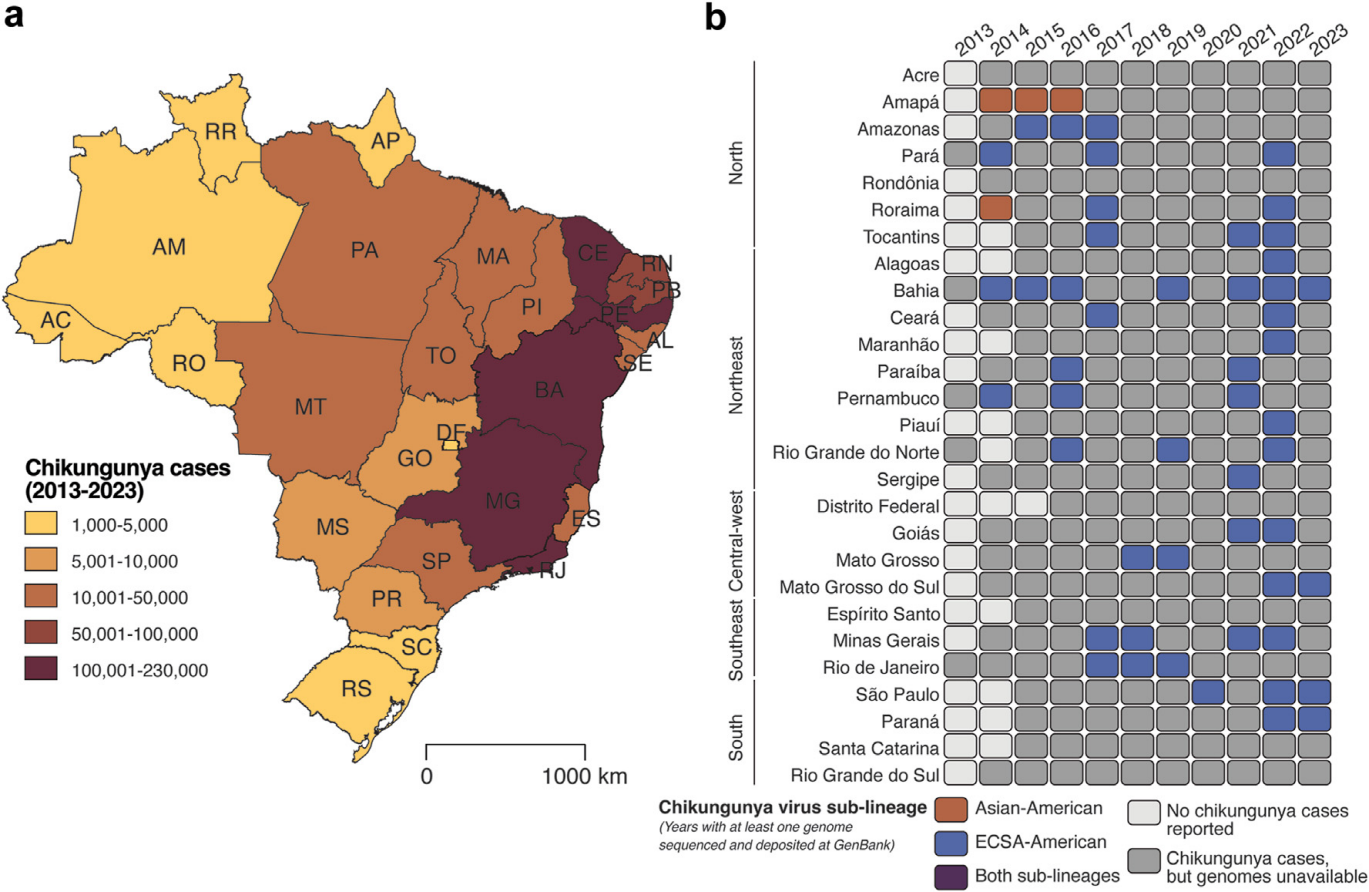
**Epidemiology and genomic information of chikungunya in Brazil. (a)** Map shows cumulative chikungunya cases (i.e., suspected and laboratory-confirmed). In all 26 Brazilian States and the Federal District reported to the Brazilian Ministry of Health from March 2013 to June 2023. **(b)** Spatiotemporal distribution of chikungunya virus lineages in countries and territories in all 26 Brazilian States and the Federal District. The chikungunya lineage circulating was determined based on years with at least one genome sequenced and deposited in GenBank through 17 August 2023. AC = Acre. AL = Alagoas. AM = Amazonas. AP = Amapá. BA = Bahia. CE = Ceará. ES = Espírito Santo. DF = Distrito Federal (Federal District). GO = Goiás. MA = Maranhão. MG = Minas Gerais. MS = Mato Grosso do Sul. MT = Mato Grosso. PA = Pará. PB = Paraíba. PE = Pernambuco. PI = Piauí. PR = Paraná. RJ = Rio de Janeiro. RN = Rio Grande do Norte. RO = Rondônia. RR = Roraima. RS = Rio Grande do Sul. SC = Santa Catarina. SE = Sergipe. SP = São Paulo. TO = Tocantins. Km = kilometers. ECSA-American, East-Central-South-African-American sub-lineage.

In August 2014, local transmission of the Asian-American CHIKV sub-lineage and of a novel ECSA sub-lineage (i.e., ECSA-American) was detected in Brazil, in Oiapoque City (Amapá State) and Feira de Santana City (Bahia State), respectively.^12^ The presumed index case infected with the ECSA-American CHIKV was an individual who had returned from Angola.^30^ An Angolan origin of the ECSA-American CHIKV sub-lineage is also supported by the close genetic similarity to an ECSA CHIKV strain described in Angola.^12^ In recent years, the ECSA-American sub-lineage became dominant across all Brazilian geographic regions. It was also detected in Haiti and during the 2023 epidemics in Argentina, Uruguay, and Paraguay (Fig. 2c).^31^^,^^32^ Conversely, the Asian-American sub-lineage was likely introduced into Brazil from French Guiana, and its detection in Brazil has been restricted to Amapá and Roraima States, in the North, which border French Guiana and Venezuela and Guiana, respectively (Fig. 4).^12^^,^^33^

The Asian-American and the ECSA-American CHIKV sub-lineages appear to have distinct epidemiological trajectories in the Americas. The former has not been reported since 2018 in the region, while the ECSA-American sub-lineage continues to cause large outbreaks across Brazil, Uruguay, Paraguay, and Argentina (Fig. 2a–c). Nevertheless, the sub-lineage associated with recent chikungunya cases in Bolivia, Peru, Belize, Guatemala, Venezuela, Uruguay, El Salvador, Colombia, Costa Rica, Nicaragua, and Honduras remain to be determined (Fig. 2c).

A combination of factors could explain the persistence of the ECSA-American CHIKV sub-lineage across Brazil and the South Cone region. First, local transmission of this sub-lineage was initially reported in Feira de Santana (619,609 inhabitants),^12^ the second most populated municipality in Bahia State (14.9 million inhabitants), which is the fourth-largest Brazilian State in population and the fifth-largest in area. Bahia State is an important tourism destination in Brazil, with over 6 million visitors annually, which may have facilitated the spread of the ECSA-American sub-lineage across the country. In contrast, the limited human mobility connectivity of Oiapoque (23,482 inhabitants) in the North of Brazil, where local cases of the Asian-American sub-lineage were first detected in 2014,^12^ likely limited spread of this sub-lineage to other Brazilian states. In addition to the possible roles of these ecological and demographic factors, the apparently greater virulence of ECSA strains^28^ could reflect higher levels of viremia and more efficient mosquito transmission, as well as the overall fitness superiority of this lineage based on differences in the 3’ untranslated genome region that affect overall fitness and probably reflect founder effects.^34^

Mosquito species-specific viral factors may also influence the transmission efficiency of different CHIKV lineages. For example, experimental vector competence studies suggest greater infectivity of some CHIKV ECSA strains for *Ae. albopictus* compared to *Ae. aegypti*, which is the main CHIKV vector in Brazil.^35^ However, the role of *Ae. albopictus* in CHIKV spread in Brazil remains unclear, as vector competence is but one factor in the more epidemiologically relevant vectorial capacity. Additionally, the genomic diversity of *Aedes* spp. populations across the Americas is complex; its link with different aspects of vector bionomics, including vector competence, is an important area for future research. Finally, ECSA-American sub-lineage strains could be associated with higher levels of human viremia. Future research is needed to characterize CHIKV genome-wide mutations across viral lineages and sub-lineages for viral phenotype, both in human and mosquito populations, particularly regarding transmissibility and virulence.

## Seroprevalence, severe manifestations, and deaths caused by chikungunya

CHIKV infection has been shown to elicit lifelong immune protection, which prevents disease and also probably reinfection.^7^ Therefore, determining seropositivity rates is critical to developing countermeasures and prioritizing future areas for deployment; however, CHIKV serosurveys remain patchy in the Americas. A routine, comprehensive, high-resolution, and multiple spatial scale seroprevalence study could improve the understanding of attack rates in vulnerable populations. Seroprevalence across 30 neighborhoods in Feira de Santana (Bahia, Brazil), where CHIKV ECSA-American sub-lineage was first detected in 2014^12^ varied between 2% and 70.5%.^36^ Seroprevalence was similar across age groups but highly heterogenous across neighborhoods, being lower in high-income neighborhoods, and higher in women and areas with the highest population density.^36^ Additionally, a recent systematic review of seroprevalence studies showed highly heterogeneous seropositivity, with pooled estimates from six studies in Brazil ranging from 7.4% to 51%, and between 13.1% and 57.9% in other countries and territories in the Americas.^37^ To improve insights on CHIKV seroprevalence and attack rates, serosurveys should be routinely conducted using blood bank donor samples to complement traditional pathogen detection and sequencing. Moreover, serosurveys could help to elucidate the potential impact of cross-protection between CHIKV and Mayaro virus, an antigenically related arthritogenic alphavirus endemic in the Amazon region.^38^^,^^39^

CHIKV infection has been linked with diverse and severe clinical manifestations in addition to fever accompanied by arthralgia, such as central nervous system infection, cardiovascular disease, renal impairment, and deaths in Brazil, Colombia, Guadeloupe, and Puerto Rico.^3^^,^^4^^,^^40^, ^41^, ^42^ These severe manifestations have been reported in countries with a predominance of either the Asian or the ECSA lineages (Fig. 2a), although the Asian lineage appears to be less virulent.^28^ However, further work on the viral genetic factors associated with severe disease is needed because the frequency of different lineages may affect the prevalence of these clinical outcomes. In addition, inexpensive, point-of-care chikungunya diagnostics are required for proper clinical management, particularly considering areas with co-circulation of arboviruses with overlapping clinical syndromes. For example, while aspirin and other non-steroidal anti-inflammatory drugs (e.g., ibuprofen) can be useful in controlling fever and joint pain in patients with chikungunya, they can increase the risk of hemorrhagic manifestations in patients with dengue, making differential diagnosis of considerable importance in areas of co-circulation.

Estimates of chikungunya deaths vary throughout the Americas, with case-fatality rates ranging between 0.5 and 1.3 deaths per 1000.^11^^,^^43^ Several factors that might affect the risk of death can differ across countries, including surveillance and healthcare system capacity, comorbidities (e.g., hypertension, diabetes, or obesity), and population demographics, with neonates and persons >65 years of age showing higher risk compared to other ages.^3^^,^^41^ In addition, establishing an accurate surveillance system for chikungunya deaths in the Americas is challenging partly due to resource limitations and the co-occurrence of other fatal arboviral diseases with similar clinical manifestations, particularly dengue. Chikungunya deaths in cases without laboratory confirmation might be incorrectly attributed to other etiologies because the clinical presentation of fatal chikungunya is undistinguishable from that of other febrile illnesses, such as dengue, which are incorrectly assumed to have a higher risk of fatal outcome. To better estimate its burden, chikungunya should be considered as a possible cause of death in endemic countries. Also, mechanisms and potential biomarkers associated with neurologic involvement, chronic disease, and fatal outcomes must be elucidated to support the development of antiviral therapies, improve clinical management, and reduce the burden imposed by severe chikungunya.

## Chikungunya in the Americas: a call for action

A cohesive, strategic, and timely plan is needed to mitigate the disease and economic burden and possibly eliminate chikungunya from the Americas. Some steps are key in this pathway (Box 1). First, continued molecular surveillance, prompt diagnosis, and the timely and appropriate treatment of chikungunya and other mosquito-borne diseases can help reduce transmission, and improve patient outcomes. Second, modeling of genomic and serological data could help monitor CHIKV evolution and spread, and identify the proportion of susceptible populations and determine areas at risk for future epidemics. This approach should be supported by local capacity strengthening and a FAIR (Findable, Accessible, Interoperable, and Reusable) framework that explicitly promotes equity.^21^ Third, a better understanding of the spatiotemporal transmission dynamics of CHIKV at multiple geographical scales (municipality, state/department, and national levels) is critical for improving mitigation strategies, including during inter-epidemic periods. Fourth, novel approaches for vector control and reducing vectorial capacity for CHIKV transmission are urgently needed because the current strategies have been ineffective, expensive, and environmentally problematic. For example, the release of *Ae. aegypti* infected with Wolbachia (*wMel* strain) in Rio de Janeiro city was spatially correlated with fewer dengue and chikungunya cases, presumably due to reductions in vector competence caused by these bacterial symbionts.^44^ Singapore has also successfully implemented a control and prevention program combining traditional and new technologies (e.g., Gravitrap and *Wolbachia*-infected male mosquitoes)^45^; thus, large-scale deployments based on this experience could be better explored in the Americas. There, after the World Mosquito Program (WMP) introduced *Wolbachia*-infected mosquitoes in Colombian cities, the dengue incidence dropped by 94–97%.^46^ Now, WMP plans to scale up *Wolbachia* program in Brazilian urban areas over the next decade, with the goal of to protecting up to 70 million people from mosquito-borne diseases like CHIKV, ZIKV, and DENV.^46^ Fifth, efficient, affordable, and licensed vaccines for most at-risk populations are essential to reduce CHIKV transmission and disease. In recent years, several chikungunya vaccine candidates have been shown to be safe and immunogenic, including with a single-dose regimen.^47^^,^^48^ Following the recent regulatory approval of the first effective and safe chikungunya vaccine,^5^ prioritizing most at-risk populations with limited supplies will be key to maximize success of forthcoming immunization programs. Because CHIKV infection appears to provide long-life immune protection, even against heterologous viral lineages,^7^^,^^49^ chikungunya could potentially be eliminated in the Americas, assuming that the virus does not spill back into an enzootic American cycle, as occurred centuries ago for yellow fever virus.^50^ Therefore, continuous monitoring of potential CHIKV spillback within a One Health approach, combined with surveilling and controlling human-amplified transmission in regions with large nonhuman primate populations, will be important to assess the feasibility of chikungunya eradication in the Americas.

Box 1 **Key proposed actions to mitigate and eliminate chikungunya in the Americas.**

• Enhance molecular surveillance and diagnosis of chikungunya cases to track endemic transmission and outbreaks and improve patient outcomes effectively.
• Leverage genomic and serological data to identify susceptible populations, anticipate future outbreaks, and monitor chikungunya virus evolution and transmission.
• Identify environmental and socioeconomic drivers of chikungunya spatiotemporal transmission during and between outbreaks to refine mitigation strategies.
• Deploy cutting-edge and traditional vector control technologies to reduce the population of competent vectors and chikungunya incidence.
• Implement immunization programs in vulnerable populations to curb and potentially eliminate chikungunya transmission across the Americas.
• Surveillance of non-human primate populations located in proximity to human settlements is crucial to track potential transmission from humans to wild animals (i.e., spillback).

## Contributors

WMdS, GSR, NRF, and SCW were responsible for conceptualizing the viewpoint and writing the first draft; MAS, UK, CVFC, ECS, NRF, and SCW performed critical revisions of the viewpoint and contributed to the writing. STSdL, RdJ, FRRM, and CW contribute to compiling data, interpretation, and discussion. All authors reviewed and agreed on the final version of the viewpoint.

## Editor's note

The Lancet Group takes a neutral position with respect to territorial claims in published maps and institutional affiliations.

## Declaration of interests

The authors declare no conflict of interest.
